# Quality of Online Information on Multiple Myeloma Available for Laypersons

**DOI:** 10.3390/curroncol29070358

**Published:** 2022-06-27

**Authors:** Henrike Staemmler, Sandra Sauer, Emma Pauline Kreutzer, Juliane Brandt, Karin Jordan, Michael Kreuter, Mark Kriegsmann, Hartmut Goldschmidt, Carsten Müller-Tidow, Gerlinde Egerer, Katharina Kriegsmann

**Affiliations:** 1 Department of Hematology, Oncology and Rheumatology, University Hospital Heidelberg, Im Neuenheimer Feld 410/69120, 69120 Heidelberg, Germany; henrike.staemmler@web.de (H.S.); sandra.sauer@med.uni-heidelberg.de (S.S.); emmapauline.kreutzer@gmail.com (E.P.K.); juliane.brandt@med.uni-heidelberg.de (J.B.); karin.jordan@med.uni-heidelberg.de (K.J.); hartmut.goldschmidt@med.uni-heidelberg.de (H.G.); carste.mueller-tidow@med.uni-heidelberg.de (C.M.-T.); gerlinde.egerer@med.uni-heidelberg.de (G.E.); 2 Center for Interstitial and Rare Lung Diseases, Department of Pneumology, Thoraxklinik, University Hospital Heidelberg, 69126 Heidelberg, Germany; michael.kreuter@med.uni-heidelberg.de; 3 German Center for Lung Research (DZL), 69120 Heidelberg, Germany; mark.kriegsmann@med.uni-heidelberg.de; 4 Institute of Pathology, University Hospital Heidelberg, 69120 Heidelberg, Germany; 5 National Center for Tumor Diseases (NCT), University Hospital Heidelberg, 69120 Heidelberg, Germany

**Keywords:** multiple myeloma, online health information, patient education

## Abstract

Online information can increase patients’ competence and engagement. However, there are concerns regarding invalid information. Overall, 300 websites and 50 YouTube videos on multiple myeloma (MM) were evaluated. The websites did not differ between the search engines or search ranks. The median time since the last update was 9 months. The 63 unique websites showed a poor general quality (median JAMA score 2 of 4, only 18% with a valid HON certificate). The patient- (user-) focused quality was medium to poor (median sum DISCERN score 41 out of 80 points). The overall reading level was difficult requiring at least a 12th US school grade. The content level was low (median 24 out of 73 points). Sixteen percent contained misleading/wrong facts. Websites provided by foundation/advocacies showed a significantly higher general and patient- (user-) focused quality. For videos, the median time since upload was 18 months. Judged by the HON foundation score ~80% of videos showed a medium general quality. The patient- (user-) focused quality was medium to poor (median sum DISCERN score 43 points). The content level was very low (median 8 points). MM relevant websites and videos showed a medium to low general, patient- (user-) focused and content quality. Therefore, incorporation of quality indices and regular review is warranted.

## 1. Introduction

An increasing number of online information sources on virtually all health-related topics and diseases are available and easily accessible to patients and other interested users. The variety of the online sources published on or delivered by the World Wide Web is enormous: websites provided by academic or non-academic institutions, social media, blogs and support forums, patient portals, news aggregators, etc. [1]. The 2019 survey by Eurostat, the statistical office of the European Union, revealed that “one in two citizens (53%) aged 16–74 reported that they sought online health information related to injury, disease, nutrition, improving health or similar” during the last three months [2].

As shown by previous analyses, online information gathering can increase patients’ competence with and engagement in health decision-making strategies and health maintenance [3,4,5]. Despite these advantages, there are several concerns regarding an overabundance of irrelevant, incomplete, or invalid information, anxiety and poor compliance resulting from a false interpretation of written information, destruction by unrelated information, and selective patients’ perception that satisfies the search intention [6,7,8,9,10]. One might argue, that high-quality diagnostic and clinical guidelines and academic articles are also available online. However, these sources of information are not freely available and are written at a high reading level, which negatively influences accessibility.

Multiple Myeloma (MM) is a malignant plasma cell (PC) neoplasm “that accounts for 1–1.8% of all cancers and is the second most common hematological malignancy” [11]. Despite the availability of novel, highly effective agents and elaborated therapy stages “only 10–15% of patients achieve or exceed expected survival compared with the matched general population” [11,12]. This implicates a long-lasting physician-patient-relationship and informed decision-making processes, particularly regarding aggressive therapy regimens, such as high-dose chemotherapy, and treatment options in case of progressive disease. During the course of the disease patients and their relatives often seek for MM related information online and address it during the medical consultation. Therefore, knowing what content is assessable for patients may help clinicians to educate their patients and actively address misinformation [13].

The aim of the current analysis was to evaluate the quality of online resources available for MM, therefore addressing the risk of misinformation by insufficient, incorrect, misleading, and out-of-date information. This was achieved by a score-based assessment of general and patient-focused quality, readability, and content of websites and YouTube videos.

## 2. Methods

### 2.1. Website and Video Search and Selection Strategy

Written online health information on MM in English using the three most common internet search engines, i.e., Google, Bing, and Yahoo, were analyzed. US American version for the search engines was used. Searches were performed from 24 February–16 March 2020 after the removal of the web browser cookies and history. The search term “multiple myeloma” was applied. The first 100 search hits in each search engine were selected for further evaluation, resulting in 300 overall initial websites.

To identify videos with health information on MM the online video-sharing platform YouTube was searched for the term “multiple myeloma”. Searches were performed from 30 March–2 April 2020. The first 50 search hits were selected for further evaluation.

Websites and videos with no relevance to MM (f. e. only including the search term in a bullet list), requiring fees or registration, using other languages than English, or scientific journal articles intended for professionals were defined as not eligible.

For website evaluation, in a first step, websites not meeting the eligibility criteria and duplicates were excluded for each search engine separately and an evaluation by the search engine was performed. In a second step, the final overall list of websites was created by removing the duplicates between the search engines. These websites (called “unique”) were meant to represent the primarily assessable body of information to the patients and were subjected to an elaborate analysis.

In the case of videos an evaluation regarding different search platforms was not performed, as only YouTube was used.

### 2.2. Assessed Variables and Scores

For detailed evaluation, general website and video information, the general quality of medical information online, patient- (user-) focused quality of medical information online, readability, and entity-related content were assessed (Table S1) [14]. We previously published an evaluation of websites and videos on monoclonal gammopathy of undetermined significance using the same methodological approach [15].

*General information* on websites included the search rank, paid advertising website (yes versus no), URL (Uniform Resource Locator), host continent, website category (scientific/governmental, foundation/advocacy f. e. American Cancer Society or International Myeloma Foundation, news/media, industry/for-profit, personal commentary/blog), update and access date.

General information on videos included the search rank, URL/title, host continent, video category (academic institution, governmental organization, news/media, industry/for-profit organization, independent medical professional, independent non-medical user), upload and access date, video duration, number of views, likes, dislikes, and comments. The viewing rate was calculated as: views/days since upload. The engagement rate was calculated as: (likes + dislikes + comments)/views.

The *general quality of medical information online* was evaluated by the Health on the Net (HON) foundation certificate/score and the Journal of the American Medical Association (JAMA) score. HON is an international not-for-profit, non-governmental organization that promotes transparent and reliable health information online. Providers of health information websites can certify their website by the HON foundation [16,17]. As the HON certificate applies to websites only, a step-by-step evaluation of videos was performed according to the eight HON foundation principle criteria (authority, complementarity, confidentiality, attribution, justifiability, transparency of authorship, transparency of sponsorship, honesty in advertising, and editorial policy; minimum points 0, maximum points 8). The achieved points translate into a low (0–2 points), medium (3–5 points), and high (6–8 points) quality category according to the HON foundation. The JAMA score evaluates a series of four criteria (authorship, attribution, disclosure, and currency; minimum points 0, maximum points 4) and aims to assess, control, and assure the quality of medical information on the internet [18].

The *patient- (user-) focused quality of medical information online* was evaluated by the DISCERN score. The DISCERN score assesses the quality of written consumer health information on treatment choices. It addresses the following questions: is the publication reliable? (Section 1, items 1–8); how good is the quality of information on treatment choices? (Section 2, item 9–15); overall rating of the publication (item 16) [19]. A minimum of 1 (not addressed) and a maximum of 5 points (fully addressed) can be achieved per DISCERN score item, resulting in a minimum of 16 (1 point × 16 items) and a maximum of 80 (5 points × 16 items) achievable points per website/video. To ensure the best possible objectivity, the evaluation of websites and videos according to the DISCERN was performed by two observers.

The *readability* of websites was assessed according to the Flesch Reading Ease score and the Flesch Kincaid Grade level. The Flesch Reading Ease score was calculated as: 206.835 − (1.015 × average sentence length) − (84.6 × average number of syllables per word), and ranges between 0–30 (a text very difficult to understand) and 90–100 (a text very easy to understand) [20]. The Flesch Kincaid Grade level uses a modified Flesch Reading Ease formula ((0.39 × average sentence length) + (11.8 × average number of syllables per word) − 15.59) to produce a grade-level score according to the US school grade that is needed to understand a text [21].

The *entity-related content* was assessed according to 73 key facts on MM derived from current guidelines and addressing the categories definition, symptoms, risk factors, evaluation, prognostic factors, management, supportive care, and outcome (Table S2) [11,22,23]. Points that were achievable per key fact items included 0 (not addressed), 0.5 (partially addressed), and 1 (fully addressed), resulting in a minimum of 0 (0 points × 73 items) and a maximum of 73 (1 point × 73 items) achievable overall content points per website/video. If applicable, misleading and wrong facts were recorded and classified according to the key fact category.

### 2.3. Statistical Analysis

Statistical analysis was performed in R studio (R version 4.0.2, 22 June 2020, The R Foundation for Statistical Computing). Data were presented as absolute numbers and percentages, medians and ranges as well as means and standard deviations (SD) as appropriate.

To analyze contingency tables Fisher’s exact was used. The distribution of ordinal scaled variables was compared by the Mann–Whitney U test (two groups) or the Kruskal–Wallis H test (more than two groups). To identify differences between group means for continuous variables, comparisons were performed with unpaired two-tailed Student’s *t*-tests (two groups) or analysis of variance (ANOVA, more than two groups). Linear regression was performed to investigate the correlation between the website search rank and the sum DISCERN score, sum key facts score, and time since update.

Inverse Kaplan-Meier curves were chosen to demonstrate the proportion of website/video updates by time. An event represents a website update. The time to event was calculated as a difference in months between the update/upload and access date. Websites not indicating an update date were excluded from Kaplan-Meier curve representation.

Unsupervised hierarchical clustering (R package ‘ComplexHeatmap’) by the website and DISCERN or key fact score items was performed in order to identify an association between website category and addressed items.

A *p*-value of ≤ 0.05 was considered statistically significant.

## 3. Results

### 3.1. Characterization of Websites on MM

#### 3.1.1. Search and Selection Results

The initial search resulted in 300 overall websites for the three search engines; 53, 38, and 23 websites met the eligibility criteria for Google, Bing, and Yahoo, respectively. The removal of duplicates between the three search engines resulted in 63 unique MM relevant websites (Table S3A).

#### 3.1.2. Characteristics According to Search Engine

In order to identify differences between the three search engines a characterization and comparison of websites found on Google (*n* = 53), Bing (*n* = 38), and Yahoo (*n* = 23) was performed (Supplementary Table S4).

We could not identify any statistically significant differences between the general website information, i.e., website category, and host continent. There were nine promoted websites (i.e., paid advertising). A comparison of websites with an indicated update date regarding the time since last update did not show any statistically significant differences (median time since last website update: Google 9 months, Bing 9 months, Yahoo 8 months, *p* = 0.313, Figure S1D).

The MM relevant websites identified on Google, Bing and Yahoo also did not significantly differ in terms of general quality of medical information online (availability of the HON foundation certificate, JAMA score), patient- (user-) focused quality of medical information online (DISCERN score), readability (Flesch Reading Ease score, Flesch Kincaid Grade Level), and MM related content (key fact score, misleading/wrong facts).

**Figure 1:**
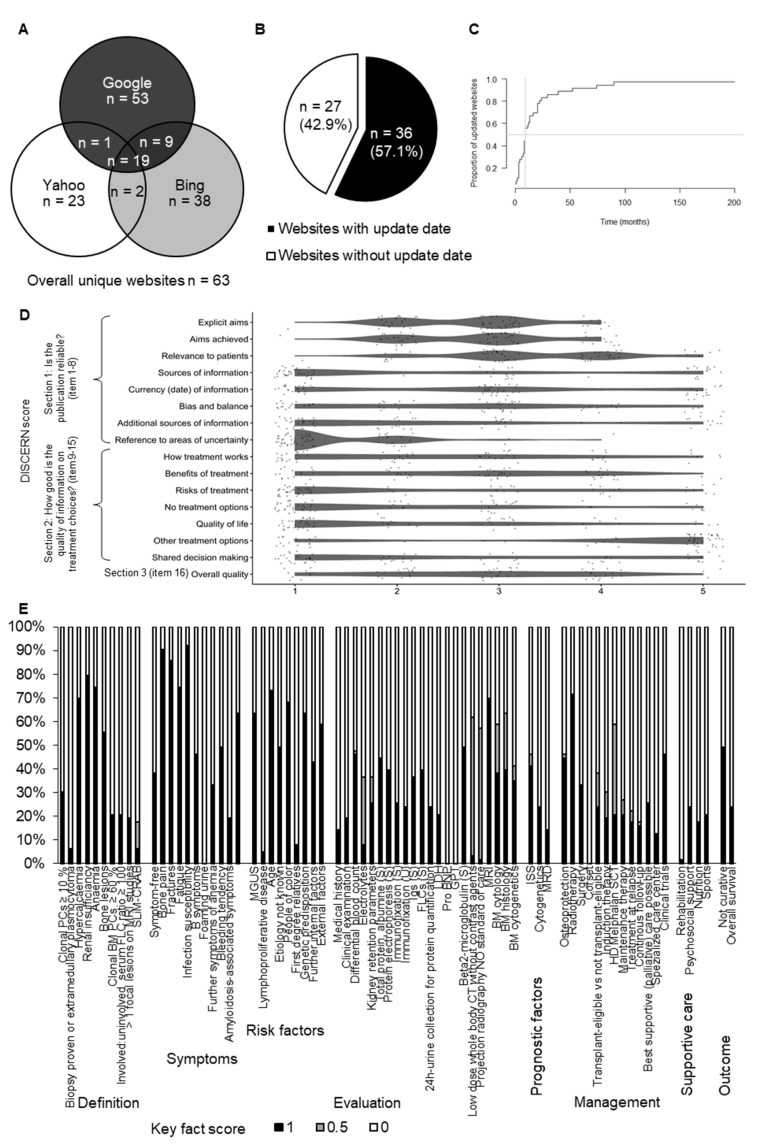
Characterization of unique websites. (**A**) Venn diagram indicating the number of duplicate overlaps between the search engines. Overall 63 unique websites were identified. (**B**) Pie chart showing the proportion of websites with and without indicated update date. (**C**) The inverse Kaplan-Meier curve shows the proportion of website updates by time. Only websites with an indicated update date were included (*n* = 36). (**D**) A scatter dot plot shows the score result reached by every single website (*n* = 63) for each item of the DISCERN score. The categorial item scoring ranges between 1 (not addressed/fulfilled) and 5 (fully addressed/fulfilled). To avoid a visual overlap the dots were spread around the respective score category. (**E**) For each of the 73 key fact items, the proportion of websites (*n* = 63) fully (1), partially (0.5) or not (0) addressing the respective contents is shown. The results are grouped by key fact category.

Moreover, no correlation between the search rank and the sum of the DISCERN score, the sum of the key fact score, or the time since upload was identified for any search engine (Figure 1A–C).

#### 3.1.3. Quality and Content of Unique Websites

Sixty-three unique MM relevant websites, representing the primarily assessable body of information to the patients, were identified (Figure 1A) and subjected to an elaborate characterization (Table 1).

The most websites were provided either by a foundation/advocacy (*n* = 31, 49.2%) or an industry/for profit organization (*n* = 20, 31.7%). Eleven websites on MM (17.5%) related to news/media and only one website (1.6%) was provided by a scientific/governmental institution. All but one website originated from North America (*n* = 62, 98.4%). 36 websites (57.1%) indicated an upload/update date (Figure 1B). The median time since upload/last update of these websites was 9 months (Figure 1C).

The general quality of medical information online was low according to the JAMA score: of four maximum achievable points on authorship, attribution, disclosure, and currency, in median 2 (range 1–4) points were met by the evaluated websites. Moreover, only a few websites had a valid HON foundation certificate (*n* = 11, 17.5%).

**Figure 2:**
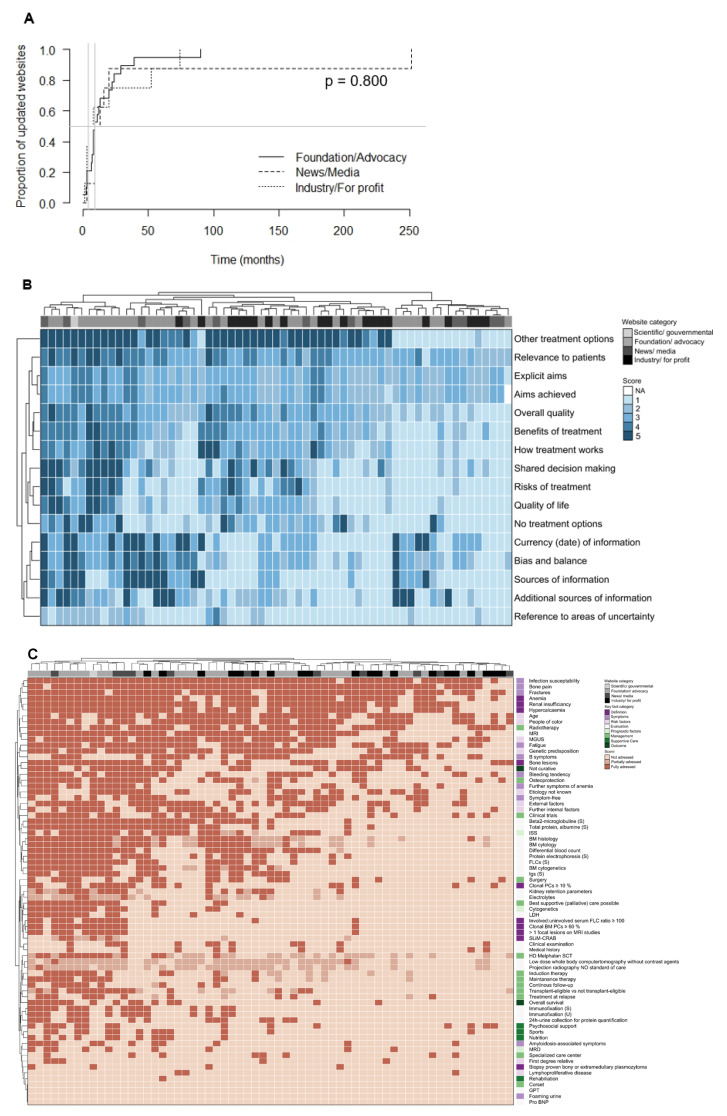
Characterization of websites by category. (**A**) Inverse Kaplan-Meier curves show the proportion of website updates by time and website category. Only websites with an indicated update date were included (foundation/advocacy *n* = 19, news/media *n* = 8, industry/for profit *n* = 8). The available scientific/governmental website (*n* = 1) was excluded from the analysis. (**B**) Unsupervised hierarchical clustering was performed by DISCERN score items (rows) and single websites (columns, *n* = 63). Item 2. “aims achieved” displays not assessable (NA) scores, as it was only assessable if item 1. “explicit aims” was not scored with 1. The website category is shown in the top row of the heatmap. Websites provided by foundations/advocacies cluster on the left side of the heat map, indicating a higher number of addressed DISCERN score items. (**C**) Unsupervised hierarchical clustering was performed by key fact score items (rows) and single websites (columns, *n* = 63). The website category is shown in the top row and the key fact item category is in the last column of the heatmap. Websites provided by foundations/advocacies cluster on the left side of the heat map, indicating a higher number of addressed key facts.

The patient- (user-) focused quality of medical information online assessed by the DISCERN score was intermediate: out of 16 minimum and 80 maximum possible points, in median 41 (range 17–68) points were achieved by the evaluated websites. The Section 1 (reliability of information) items “sources of information” and “references to areas of uncertainty” were poorly addressed. In Section 2 (quality of information on treatment choices) particularly the item “risk of treatment” item, i.e., side effects of treatment, were not addressed by most websites. Contrary, treatment options were discussed by most websites (Figure 1D and Figure 2B, Table S5A).

The readability according to the mean Flesch Reading Ease score of the evaluated websites was difficult (mean 40, SD 10). The mean Flesch Kincaid Grade level was 12 (SD 2), indicating that at least a 12th US school grade is required to understand the content.

The MM-related content was evaluated by a set of 73 very key facts (Figure 1E and Figure 2C). Out of 73 maximum possible points, in median 24 (range 4–54) points were achieved. Regarding the definition of MM diagnosis, CRAB criteria (hypercalcaemia, renal insufficiency, anemia, bone lesions) were addressed in >50% of websites. However, the recently introduced SLiM criteria (clonal bone marrow [BM] PCs ≥ 60%, involved/uninvolved serum free light chain ratio ≥100, >1 focal lesions on magnetic resonance imaging) were mentioned in less than one-third of websites. Except for lymphoproliferative disease and first-degree relative, risk factors of MM were covered by over 40% of the evaluated websites. Regarding the MM evaluation, assessment of medical history and clinical examination were addressed in 10–20% of websites. Information on the evaluation of MM activity by monoclonal protein assessment in serum and urine, electrolytes, and kidney retention parameters was provided by approximately 30% of websites. Assessments of liver (GPT, glutamate pyruvate transaminase) and heart function (pro-BNP, pro brain natriuretic peptide) were not addressed. Imaging and BM diagnostics were covered by approximately 50–60% of websites. Prognostic factors and treatment options were mentioned on approximately 20–30% of websites. MM being a not curable disease was addressed by 50% of websites.

Ten websites (15.9%) containing overall fifteen misleading/wrong facts were identified (Table S6A). Three misleading/wrong facts (20.0%) were related to the definition of MM diagnosis. Exemplarily, for MM diagnosis a threshold of 30% BM PCs was falsely indicated. Also, misleading information on risk factors (*n* = 1, 6.7%), evaluation (*n* = 2, 13.3%), prognostic factors (*n* = 1, 6.7%), and management (*n* = 8, 53.3%) of MM was provided. Moreover, not state-of-the-art treatment options were indicated.

A data set containing the original data on selected quality, content scores, and citations of misleading/wrong facts obtained from the unique websites is provided along with this manuscript (Table S7).

#### 3.1.4. Website Characteristics According to Category

In order to identify differences between the website sources, a characterization and comparison of websites by providers was performed (foundation/advocacy *n* = 31, news/media *n* = 11, industry/for profit *n* = 20, Table 2). Due to a low case number, the scientific/governmental category (*n* = 1) was not included.

Although, the median time since the last update was shorter in websites provided by industry/for-profit organizations (4 months) compared to foundations/advocacies (9 months) or news/media (9 months), this difference was statistically not significant (*p* = 0.800, Figure 2A).

In terms of general quality of medical information online, websites provided by industry/for profit organizations achieved a lower JAMA score (median 1, range 1–3) compared to foundations/advocacies (median 3, range 1–4) and news/media (median 3, range 1–4), (*p* = 0.001). The highest proportion of valid HON foundation certificates was found in the category news/media (*n* = 4, 36.4%), followed by foundation/advocacy (*n* = 6, 19.4%) and industry/for profit (*n* = 1, 5.0%).

The MM relevant websites of different providers did not significantly differ in terms of readability (Flesch Reading Ease score, Flesch Kincaid Grade Level).

Patient- (user-) focused quality of medical information online assessed by the DISCERN score was highest in websites provided by foundations/advocacies, followed by news/media (median 37, range 21–68) and industry/for-profit organizations (median 34, range 22–46), (global *p* = 0.009, foundation/advocacies versus industry/for profit *p* = 0.001, other group comparisons not significant, Figure 2B).

Websites provided by foundations/advocacies had the highest sum key facts score (median 26, range 9–53), followed by news/median websites (median 24, range 4–54) and industry/for-profit organizations (median 19, range 5–37), (*p* = 0.326, Figure 2C). The lowest number of websites with misleading/wrong facts was found in the category foundation/advocacy (*n* = 2, 6.5%), followed by industry/for profit and news/media with over 20% of websites with misleading/wrong facts.

### 3.2. Characterization of Videos on MM

#### 3.2.1. Search and Selection Results

50 YouTube videos were selected for initial evaluation. The removal of duplicates between the search terms and videos not meeting the eligibility criteria resulted in 45 unique videos (Table S3B).

#### 3.2.2. Quality and Content of Unique Videos

The 45 unique videos were assumed as the primarily assessable body of information and subjected to an elaborate characterization (Table 1).

**Figure 3:**
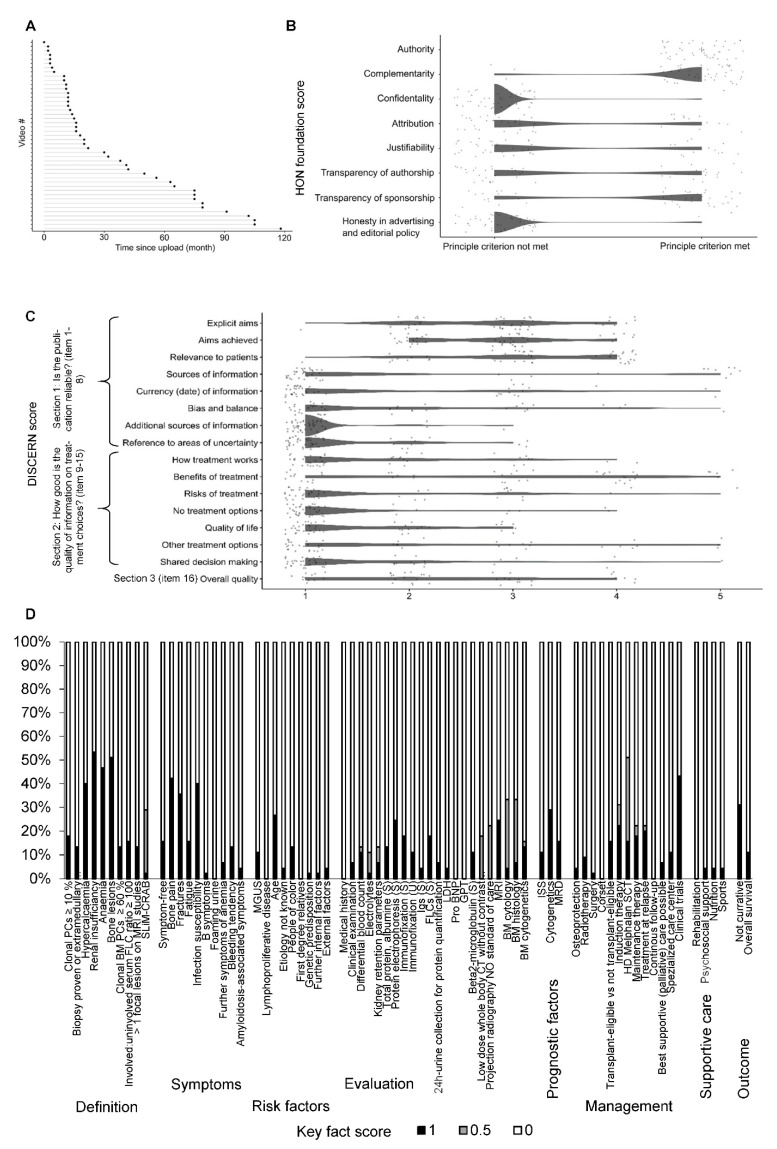
Characterization of unique videos. (**A**) The lollipop plot shows the videos ordered by their time since upload until the date of assessment in months. (**B**) A scatter dot plot shows the score result reached by every single video (*n* = 45) for each item of the HON foundation score (0, principle criterium not met; 1, principle criterium met). To avoid a visual overlap the dots were spread around the respective score category. (**C**) A scatter dot plot shows the score result reached by every single video (*n* = 45) for each item of the DISCERN score. The categorial item scoring ranges between 1 (not addressed/fulfilled) and 5 (fully addressed/fulfilled). To avoid a visual overlap the dots were spread around the respective score category. (**D**) For each of the 73 key fact items, the proportion of videos (*n* = 45) fully (1), partially (0.5) or not (0) addressing the respective contents is shown. The results are grouped by key fact category.

The most videos were provided by independent medical professionals (*n* = 22, 48.9%), followed by academic institutions (*n* = 10, 22.2%), industry/for profit organizations (*n* = 9, 20.0%) and independent non-medical users (*n* = 4, 8.9%). Most videos originated from North America (*n* = 44, 97.8%). The median time since upload of the evaluated videos was 18 months (Figure 3A). The videos had a median duration of 9 (range 1–72) minutes and 4902 (range 19–251,859) views. A median viewing rate of 6.38 (range 0.65–147.46) indicated that in median the videos were watched more than six times a day. The engagement rate was very low (median 0.01, range 0.00–0.07), resulting from low numbers of likes, dislikes, and comments.

As the JAMA score and HON foundation certificate refer to websites, the general quality of medical information online was assessed in a step-by-step evaluation of videos according to the eight HON foundation principle criteria. Out of 8 maximum points, in median 3 (range 2–6) points were achieved by the evaluated videos. While the items authority, complementarity, and transparency of sponsorship were addressed by most videos, aspects of confidentiality, attribution, justifiability, transparency of authorship, and honesty in advertising and editorial policy were generally not met (Figure 3B). A total of 5 videos (11.1%) were ranked as high, 35 videos (77.8%) as a medium, and 5 videos (11.1%) as low quality according to the HON foundation score.

**Figure 4:**
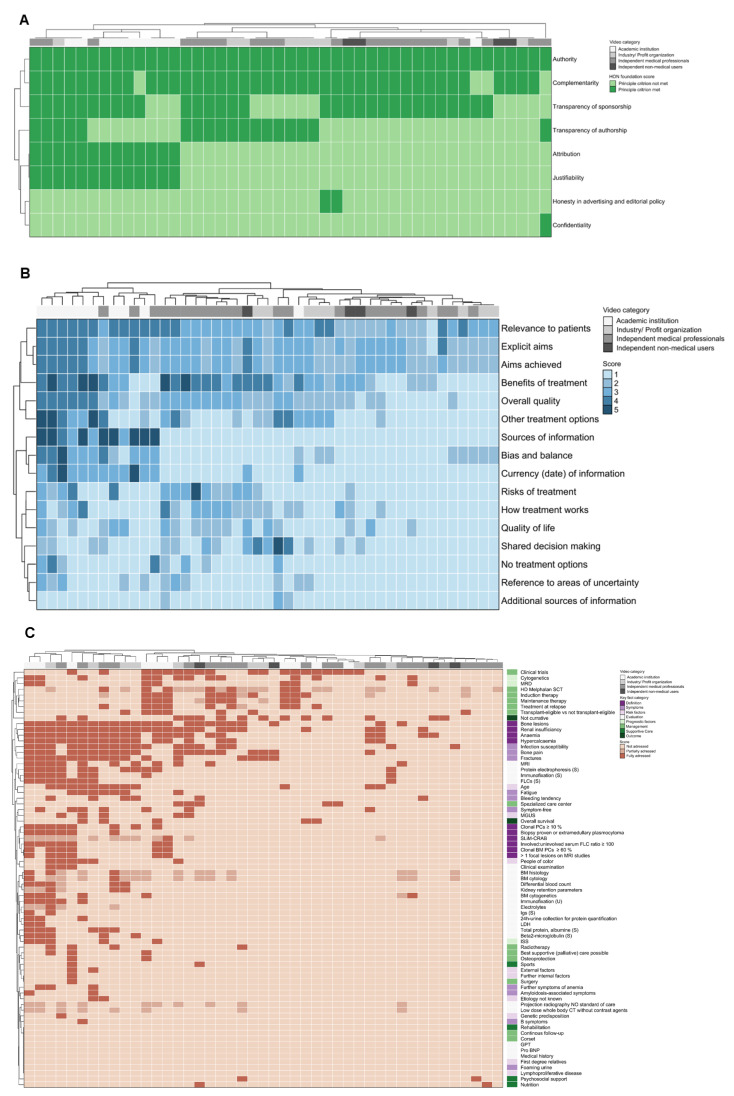
Characterization of videos by category. (**A**) Unsupervised hierarchical clustering was performed by HON foundation score items (rows) and single videos (columns, *n* = 45). The video category is shown in the top row of the heatmap. (**B**) Unsupervised hierarchical clustering was performed by DISCERN score items (rows) and single videos (columns, *n* = 45). Item 2. “aims achieved” displays not assessable (NA) scores, as it was only assessable if item 1. “explicit aims” was not scored with 1. The video category is shown in the top row of the heatmap. Websites provided by academic institutions cluster on the left side of the heat map, indicating a higher number of addressed DISCERN score items. (**C**) Unsupervised hierarchical clustering was performed by key fact score items (rows) and single videos (columns, *n* = 45). The video category is shown in the top row and the key fact item category is in the last column of the heatmap. No obvious clusters of video categories were identified.

The patient- (user-) focused quality of medical information online assessed by the DISCERN score was average: out of 16 minimum and 80 maximum possible points, in median 43 (range 26–78) points were achieved by the evaluated videos. The items “explicit aims”, “aims achieved”, and “relevance to patients” were at least addressed in most videos. However, the remaining items, including the most items on the quality of information on treatment choices (Section 2) were provided by only a few videos (Figure 3C and Figure 4B, Table S5B).

The MM-related content was evaluated by a set of 73 very detailed key facts (Figure 3D and Figure 4C). Out of 73 maximum possible points, in median 8 (range 1–35) points were achieved, demonstrating a low informational content. CRAB criteria, typical MM-related symptoms/complications such as bone pain, fractures, and infection susceptibility, as well as treatment options, such as high-dose chemotherapy and autologous stem cell transplantation, and participation in clinical trials were addressed by approximately 40–50% of videos. Other key fact items were addressed by around 30% or fewer videos.

There was one video with a misleading/wrong falsely stating that no genetic component in the etiology of MM is known (Table S6B).

A data set containing the original data on selected quality, content scores, and citations of misleading/wrong facts obtained from the unique videos is provided along with this manuscript (Table S8).

#### 3.2.3. Video Characteristics According to Category

In order to identify differences a comparison of videos by providers (academic institution *n* = 10, industry/for-profit organizations *n* = 9, independent medical professional *n* = 22) was performed (Table 3). Due to a low case number, videos provided by independent non-medical users (*n* = 4) were not included.

Videos provided by academic institutions had a longer duration (mean 36, SD 20 min), compared to videos provided by industry/for-profit organizations (mean 10, SD 8 min), or independent medical professionals (median 9, SD 9 min), (*p* < 0.001). Videos provided by industry/for-profit organizations had a higher number of views, likes, and dislikes compared to the other categories, (*p* = 0.010, *p* = 0.014, *p* = 0.040, respectively). However, the latter two did not result in a significantly higher engagement rate of industry/for-profit organization videos.

No statistically significant differences were identified between the video provider categories regarding the general quality of medical information online judged by the HON foundation score (Figure 4A) or the MM-related content assessed by the key facts (Figure 4C).

However, academic institutions provided a higher patient- (user-) focused quality of medical information online according to the DISCERN score (median 43, range 33–55), compared to independent medical professionals (median 31, range 20–42) and industry/for-profit organizations (median 26, range 18–32), (global *p* < 0.001, Figure 4B).

### 3.3. Comparison between Websites and Videos on MM

Unique websites (*n* = 63) and videos (*n* = 45) were compared regarding the DISCERN and the key fact score (Table 1).

The patient- (user-) focused quality of medical information online evaluated by the DISCERN score (minimum 16, maximum 80 points) was intermediate in both websites (median 41, range 17–68) and videos (median 43, range 26–78), with no statistically significant difference (*p* = 0.280).

The amount of MM-related content evaluated by sum key fact score (minimum 0, maximum 73 points) provided by websites (median 24, range 4–54) was higher compared to videos (median 8, range 1–35), (*p* < 0.001).

## 4. Discussion

MM patients frequently seek information related to their disease online. However, there are no previous analyses that systematically assess the general and patient-focused quality, readability, and content of websites and YouTube videos.

The characterization of selected MM-related websites can be summarized and contextualized as follows:

The website search on Google, Bing, and Yahoo revealed a substantial abundance and lack of integrity reflected by a high number of duplicates within each search engine and of websites with no relevance to MM. This significantly reduced the number of websites being eligible for the current analysis and therefore accessible to patients. The median time since the last website update was 9 (minimum 0, maximum 251) months, therefore indicating a broad range regarding the up-to-dateness. Due to rapid medical progress, the actuality of health online information is of outstanding importance [24]. The MM relevant websites did not differ in terms of analyzed scores between the three search engines or regarding their search rank. Thus, not only the search engine market leader Google, but also other search engines might be used to obtain MM-related information online [25]. Of importance, the website search rank does not necessarily correlate with the quality of MM-related online information, as a set of ranking factors is applied by search engine providers [26]. Indeed, in the current analysis, the most qualitative websites judged by the sum key fact score and sum DISCERN score were not ranked at a top position (Supplementary Table S7).

The 63 unique MM relevant websites showed a rather poor general quality of medical information online according to the JAMA score and availability of a valid HON foundation certificate (less than 20% of websites). Only a small proportion of medical and health websites provides well-established quality scores and certificates [27]. Therefore, the identification of websites that provide high-quality information is challenging, particularly for patients. Judged by the sum of the DISCERN score the patient-focused quality of MM-related information was poor to medium with therefore questionable relevance. The overall reading level on the analyzed websites was difficult and required at least a 12th US school grade to understand the website content. As “most adults read at an 8th-grade level, and 20 percent of the population reads at or below a 5th-grade level”, the MM-related website content of the analyzed websites would have been too difficult to understand for the vast majority of patients [28]. The evaluation by content key points indicated a generally low informational content level. As we applied a large set of criteria based on current diagnostic and clinical guidelines, a high sum content key fact score was not expected to be achieved by websites that are not intended for medical professionals [11,22,23]. However, 16% of websites contained misleading/wrong facts, holding a significant risk of misinformation. Websites provided by foundation/advocacies showed a significantly higher general and patient- (user-) focused quality of information online judged by the JAMA and DISCERN score, but not in MM-related content judged by key facts. Thus, showing no lower levels of MM related content, websites provided by foundation/advocacies (i.e., American Cancer Society, Canadian Cancer Society, Multiple Myeloma Research Foundation, International Myeloma Foundation, etc.) might currently represent a source of MM related information online.

The majority of previously published studies on the accuracy and reliability of internet resources, in particular websites, for information on different medical disorders and procedures mainly addressed otorhinolaryngology related topics such as sleep apnea, snoring, vestibular disorders, stuttering, and others [29,30,31,32,33,34,35,36]. Studies on the quality of online health information on other medical conditions, such as idiopathic pulmonary fibrosis, SARS-CoV-2, neurological disorders, systemic lupus erythematosus, etc., are available as well [14,37,38,39,40,41,42]. However, except for oral precancerous conditions, cancer entities were not the focus of such evaluations [43,44]. Overall, our results are in line with these previously published studies: those report on frequently outdated, mixed- or low-quality, and incomplete online health information that requires high readability skills.

The selection of MM-related videos on YouTube revealed a lower rate of abundancy (only five excluded videos) compared to websites. Given a median time since upload of 18 months, the video content can be considered outdated. Judged by the HON foundation score almost 80% of videos showed a medium general quality of medical information. Determined by the sum of the DISCERN score the patient- (user-) focused quality of MM-related information was poor to medium and therefore similar to MM-related websites. The content level according to the MM key facts was very low and therefore significantly lower compared to evaluated websites. About 50% of videos were provided by independent medical professionals and showed a significantly higher patient- (user-) focused quality of information, judged by the DISCERN score, compared to videos provided by academic institutions and industry/for-profit organizations. To the best of our knowledge, this is the first systematical and score-based evaluation of disease-related videos available on YouTube that goes beyond the assessment of provided medical content. We could identify only one “snapshot analysis of information available on youtube.com” published by Tan et al. who evaluated the quality of content regarding breast reconstruction in breast cancer patients [45]. Similar to the results obtained in the current study, the authors conclude, that the YouTube videos do not provide comprehensive information regarding the analyzed medical condition/procedure.

Beside its novelty, our study shows several methodological strengths.

A significant number of MM-related websites was analyzed upon a broad online search using the most common internet search engines, i.e., Google, Bing, and Yahoo. Going beyond written content, MM-related YouTube videos were considered. As Google and YouTube are the two most-visited websites/platforms, the selection of analyzed websites and videos might be considered representative [25]. As generally not accessed by patients, scientific journal articles intended for medical professionals were excluded from the evaluation.

The evaluation of videos and websites was performed in an objective, transparent, and reproducible score-based manner applying a set of well-established and broadly-used scores [14,29,30,31,32,33,34,35,36,38,40,41,42,43,44]. The applied set of scores did not only cover the general quality and the degree of reading difficulty, but also explicitly addressed the patient- (user-) focused quality of medical information online by the DISCERN score. Moreover, the MM-related content was extensively evaluated according to over 70 key facts derived from current guidelines covering all important aspects of MM diagnosis, treatment, and prognosis [11,22,23]. In addition, comparisons of included websites and videos regarding different aspects, such as search engine or website and video category, were performed.

The current analysis has certain limitations. These can mainly be attributed to constantly varying and potentially growing numbers, continuous updating, and changes in the rating of MM-related websites and videos. Therefore, the website and video selection in this analysis cannot be considered ultimate. Moreover, other search engines and video platforms might have led to a slightly different set of selected websites and videos.

In conclusion, the current score-based analysis of websites and videos on MM revealed an overall medium to low general, patient- (user-) focused and content quality that is provided on a high reading level. Therefore, understandability, informative value, and support in a decision-making process can be attributed to single websites/videos only. Moreover, incorporation of indices/certificates that indicate the quality of provided health information, regular review, and consideration of current guidelines of MM-related online sources is warranted.

## Figures and Tables

**Table 1:** Characterization of unique websites and videos.

Health Information Source	A. Websites		B. Videos	*p*-Value
**Overall unique websites/videos, *n* (%)**	63 (100)		45 (100)	/
**Website category, *n* (%)**		**Video category, *n* (%)**		
Scientific/governmental	1 (1.6)	Academic institution	10 (22.2)	/
Foundation/advocacy	31 (49.2)	Governmental organization	0 (0.0)	
News/media	11 (17.5)	News/media	0 (0.0)	
Industry/for profit	20 (31.7)	Industry/for profit	9 (20.0)	
Personal commentary/blog	0 (0.0)	Independent medical professional	22 (48.9)	
		Independent non-medical user	4 (8.9)	
**Host continent, *n* (%)**				
Europe	1 (1.6)		0 (0.0)	/
North America	62 (98.4)		44 (97.8)	
South America	0 (0.0)		0 (0.0)	
Asia	0 (0.0)		0 (0.0)	
Australia	0 (0.0)		1 (2.2)	
Africa	0 (0.0)		0 (0.0)	
Antarctica	0 (0.0)		0 (0.0)	
**HON foundation certificate/score**				
Valid certificate, *n* (%)	11 (17.5)		/	/
Median (range)	/		3 (2–6)	/
Top 10 websites/videos, valid certificate (*n*) or median (range)	1		3 (2–6)	/
Rating according to HON foundation score, *n* (%)				
Low	/		5 (11.1)	/
Medium	/		35 (77.8)	
High	/		5 (11.1)	
**JAMA score**				
Median (range)	2 (1–4)		/	/
**Flesch Reading Ease Score**				
Mean (SD)	40 (10)		/	/
**Flesch Kincaid Grade Level**				
Mean (SD)	12 (2)		/	/
**Video duration, minutes**				
Median (range)	/		9 (1–72)	/
Mean (SD)	/		15 (17)	/
**Views, median (range)**	/		4902 (19–251,859)	/
**Likes, median (range)**	/		36 (0–3925)	/
**Dislikes, median (range)**	/		2 (0–83)	/
**Comments, median (range)**	/		1 (0–179)	/
**Viewing rate, median (range)**	/		6.38 (0.65–147.46)	/
**Viewing rate, mean (SD)**			18.19 (31.78)	/
**Engagement rate, median (range)**	/		0.01 (0.00–0.07)	/
**Engagement rate, mean (SD)**	/		0.01 (0.01)	/
**Sum DISCERN score**				
Median (range)	41 (17–68)		43 (26–78)	0.280
Top 10 websites/videos, median (range)	46 (27–61)		26 (21–42)	/
**Sum key fact score**				
Median (range)	24 (4–54)		8 (1–35)	**<0.001**
Top 10 websites/videos, median (range)	26 (5–46.5)		15 (1–25)	/
**Misleading/wrong facts**				
Websites/videos with misleading/wrong facts, *n* (%)	10 (15.9)		1 (2.2)	/
Overall identified wrong facts, *n*	15		1	/

HON, Health on the Net; JAMA, Journal of the American Medical Association; SD, standard deviation.

**Table 2:** Characterization of websites by category.

Website Category	Foundation/ Advocacy	News/ Media	Industry/ for Profit	*p* Value
**Websites, *n***	31	11	20	
**Host continent, *n* (%)**				
Europe	1 (3.2)	0 (0.0)	0 (0.0)	/
North America	30 (96.8)	11 (100)	20 (100)	
South America	0 (0.0)	0 (0.0)	0 (0.0)	
Asia	0 (0.0)	0 (0.0)	0 (0.0)	
Australia	0 (0.0)	0 (0.0)	0 (0.0)	
Africa	0 (0.0)	0 (0.0)	0 (0.0)	
Antarctica	0 (0.0)	0 (0.0)	0 (0.0)	
**HON foundation certificate**				
Valid certificate, *n* (%)	6 (19.4)	4 (36.4)	1 (5.0)	0.087
**JAMA score**				
Median (range)	3 (1–4)	3 (1–4)	1 (1–3)	**0.001**
**Flesch Reading Ease Score**				
Mean (SD)	39 (12)	40 (8)	42 (8)	0.496
**Flesch Kincaid Grade Level**				
Mean (SD)	12 (2)	12 (1)	12 (2)	0.917
**Sum DISCERN score**				
Median (range)	45 (17–63)	37 (21–68)	34 (22–46)	**0.009**
**Sum key fact score**				
Median (range)	26 (9–53)	24 (4–54)	19 (5–37)	0.326
**Misleading/wrong facts**				
Websites with misleading/wrong facts, *n* (%)	2 (6.5)	3 (27.3)	4 (20.0)	0.169
Overall identified wrong facts, *n*	6	2	6	

As only one website was provided by a scientific/governmental institution, this website category was excluded from comparison. HON, Health on the Net; JAMA, Journal of the American Medical Association; SD, standard deviation.

**Table 3:** Characterization of videos by category.

Video Category	Academic Institution	Industry/for Profit	Independent Medical Professional	*p* Value* ^a^*	Independent Non-Medical User
**Videos, *n***	10	9	22		4
**Host continent, *n* (%)**				/	
Europe	10 (100)	9 (100)	21 (95.5)		4 (100)
North America	0 (0.0)	0 (0.0)	0 (0.0)		0 (0.0)
South America	0 (0.0)	0 (0.0)	0 (0.0)		0 (0.0)
Asia	0 (0.0)	0 (0.0)	0 (0.0)		0 (0.0)
Australia	0 (0.0)	0 (0.0)	1 (4.5)		0 (0.0)
Africa	0 (0.0)	0 (0.0)	0 (0.0)		0 (0.0)
Antarctica	0 (0.0)	0 (0.0)	0 (0.0)		0 (0.0)
Not assessable	0 (0.0)	0 (0.0)	0 (0.0)		0 (0.0)
**Video duration, minutes**					
Median (range)	38 (5–72)	9 (4–28)	6 (1–47)		3 (4–14)
Mean (SD)	36 (20)	10 (8)	9 (9)	**<0.001**	12 (18)
**Views, median (range)**	1971 (512–6083)	22,809 (1358–246,106)	2411 (19–251,859)	**0.010**	10,308 (4902–74,431)
**Likes, median (range)**	19 (4–82)	287 (15–2097)	21 (0–3925)	**0.014**	66 (46–330)
**Dislikes, median (range)**	1 (0–6)	9 (0–56)	1 (0–83)	**0.040**	7 (0–19)
**Comments, median (range)**	1 (0–9)	47 (0–136)	1 (0–179)	0.068	18 (0–62)
**Viewing rate, median (range)**	7.01 (1.60–19.25)	30.52 (2.21–123.92)	4.95 (0.65–147.46)		8.14 (2.85–23.23)
**Viewing rate, mean (SD)**	7.61 (4.90)	44.73 (44.69)	13.53 (30.71)	0.329	10.59 (9.52)
**Engagement rate, median (range)**	0.01 (0.01–0.02)	0.01 (0.00–0.03)	0.01 (0.00–0.07)		0.01 (0.01–0.02)
**Engagement rate, mean (SD)**	0.01 (0.00)	0.01 (0.01)	0.01 (0.01)	0.972	0.01 (0.01)
**HON foundation score**					
Median (range)	5 (2–6)	3 (2–6)	3 (2–6)	0.052	3 (2–3)
Rating according to HON foundation score, *n* (%)					
Low	1 (10.0)	1 (11.1)	2 (9.1)	0.934	1 (25.0)
Medium	7 (70.0)	7 (77.8)	18 (81.8)		3 (75.0)
High	2 (20.0)	1 (11.1)	2 (9.1)		0 (0.0)
**Sum DISCERN score**					
Median (range)	43 (33–55)	26 (18–32)	31 (20–42)	**<0.001**	25 (21–37)
**Sum key fact score**					
Median (range)	17 (1–33)	14 (1–35)	6 (1–25)	0.413	3 (2–12)
**Misleading/wrong facts**					
Videos with misleading/wrong facts, *n* (%)	0 (0.0)	0 (0.0)	1 (4.5)	/	0 (0.0)
Overall identified wrong facts, *n*	0	0	1		0

HON, Health on the Net; SD, standard deviation. ^a^ Due to low case number, the video category “Independent non-medical users” was excluded from the category comparison.

## Data Availability

The dataset supporting the conclusions of this article is provided as Supplementary Tables S4 and S5.

## Supplementary Materials

The following supporting information can be downloaded at: https://www.mdpi.com/article/10.3390/curroncol29070358/s1, Figure S1: Characterization of websites by search rank and time since lastthe update. The dot plots show the search rank (x axis) in dependency of the (A) sum of the DISCERN score, (B) sum of the key fact score or (C) time since last update (y axis). Each dot represents a website. The coefficient of determination R^2^ and the regression line are given for each search engine group. (D) Inverse Kaplan-Meier curves were chosen to demonstrate the proportion of website updates by time. An event represents a website update. The time to event was calculated as a difference in months between the update and access date. Only websites with an indicated update date were included (n_Google_ = 32, n_Bing_ = 25, n_Yahoo_ = 16). Table S1: Scores, measures and certificates used for the evaluation of websites and videos.; Table S2: Website and video content evaluation by MM key facts.; Table S3: Search results for websites and videos.; Table S4: Characterization of websites by search engine; Table S5: DISCERN score for websites and videos; Table S6: Misleading and wrong facts on websites and videos; Table S7: Dataset on websites; Table S8: Dataset on videos.
