# Supplementary material for: Quality of Online Information on Multiple Myeloma Available for Laypersons
Source: Curr Oncol. 2022 Jun 27;29(7):4522–40. doi: 10.3390/curroncol29070358 (PMC9324394; doi:10.3390/curroncol29070358)
Supplement: Supplementary file 1 [file curroncol-29-00358-s001.zip › Supplementary_Figure_1.pdf]

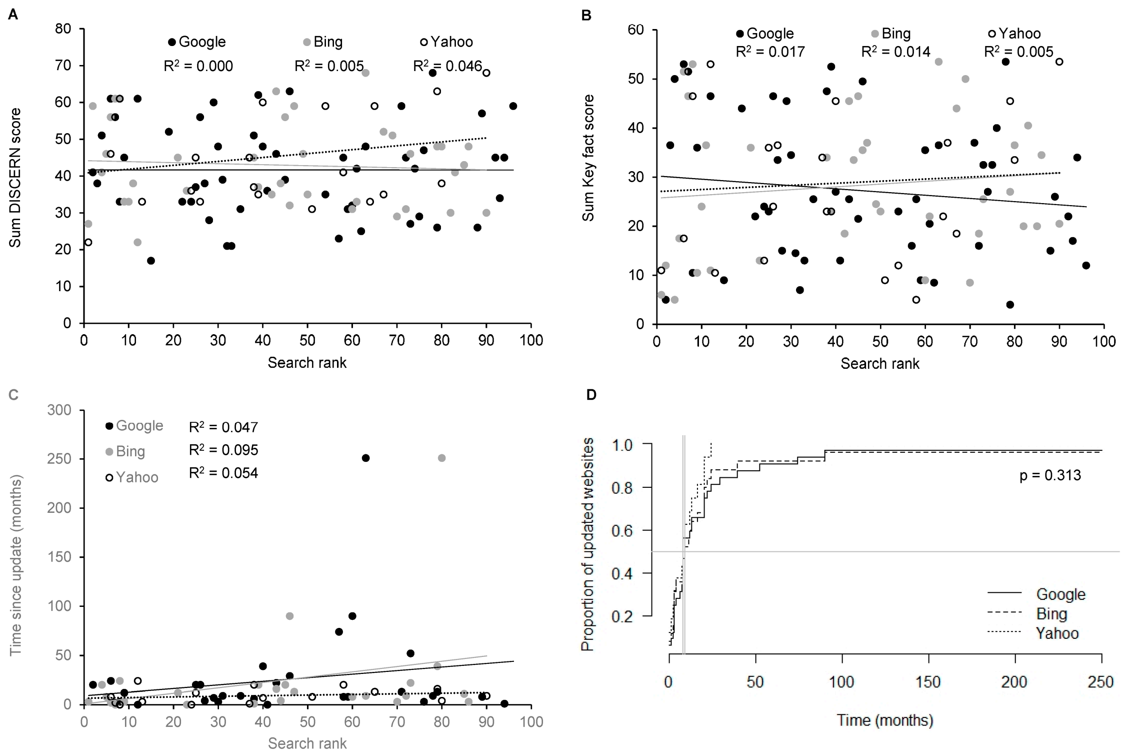

Figure S1: Characterization of websites by search rank and time since lastthe update. The dot plots show the search rank (x axis) in dependency of the (A) sum of the DISCERN score, (B) sum of the key fact score or (C) time since last update (y axis). Each dot represents a website. The coefficient of determination R2 and the regression line are given for each search engine group. (D) Inverse Kaplan-Meier curves were chosen to demonstrate the proportion of website updates by time. An event represents a website update. The time to event was calculated as a difference in months between the update and access date. Only websites with an indicated update date were included (nGoogle = 32, nBing = 25, nYahoo = 16).
