# Supplementary material for: Quality of Online Information on Multiple Myeloma Available for Laypersons
Source: Curr Oncol. 2022 Jun 27;29(7):4522–40. doi: 10.3390/curroncol29070358 (PMC9324394; doi:10.3390/curroncol29070358)
Supplement: Supplementary file 1 [file curroncol-29-00358-s001.zip › Supplementary_Table1.pdf]

Supplementary Table S1: Scores, measures and certificates used for the evaluation of websites and videos.

Score/measure/certificate	Description	Item(s)		Overall score			
		Evaluated item(s)	Scoring and description	Calculation	Scale of measure	Range	Gradation
**1. General quality of medical information online**							
**HON foundation certificate**[1, 2] Applied for website evaluation	International not-for-profit, non-governmental organization. Promotes transparent and reliable health information online. Providers of health information online can certify their website by HON foundation.	HON certification	0: HON certification not available or not valid. 1: HON certification available and valid.	/	/	/	/
**HON foundation score (detailed)** [1, 2] Applied for video evaluation	Series of eight principle criteria established by the HON foundation.	Principles of the HON code are: authority, complementarity, confidentiality, attribution, justifiability, transparency of authorship, transparency of sponsorship, honesty in advertising and editorial policy.	0: principle criterium not met 1: principle criterium met	Sum of scores for each of the 8 items.	Categorial (ordinal)	0-8	0-2: low quality 3-5: medium quality 6-8: high quality
**JAMA score**[3]	Represent a series of four criteria established by JAMA. Aims to assess, control, and assure the quality of medical information on the internet.	Authorship Attribution Disclosure Currency	0: Item criteria not met. 1: Item criteria met.	Sum of scores for each of the four items.	Categorial (ordinal)	0-4	/
**2. Patient- (user-) focused quality of medical information online**							
**DISCERN score**[4] By item	Instrument for judging the quality of written consumer health information on treatment choices. Section 1: Is the publication reliable? (item 1-8) Section 2: How good is the quality of information on treatment choices? (item 9-15) Section 3: Overall rating of the publication. (item 16)	Items: 1. explicit aims, 2. aims achieved (only applicable if item 1. is not scored with 1), 3. relevance to patients, 4. sources of information, 5. currency (date) of information, 6. bias and balance, 7. additional sources of information, 8. reference to areas of uncertainty, 9. how treatment works, 10. benefits of treatment, 11. risks of treatment, 12. no treatment options, 13. quality of life, 14. other treatment options, 15. shared decision making, 16. overall quality.	No - Partially -Yes 1 - 2 - 3 - 4 - 5	Per item	Categorial (ordinal)	1-5	/
Overall score				Sum of scores for each of the 16 items.	Categorial (ordinal)	16-80	/
**3. Readability**							

**Flesch Reading Ease Score**[5]	Measure of readability.<br>Evaluates how difficult it is to understand is a text.	/	/	Score = 206.835 - (1.015 x average sentence length) - (84.6 x average number of syllables per word)	Metric (interval)	No theoretical lower bound<br>121.22 as highest (easiest) possible score	0-30: very difficult<br>30-50: difficult<br>50-60: fairly difficult<br>60-70: standard<br>70-80: fairly easy<br>80-90: easy<br>90-100: very easy
**Flesch Kincaid Grade Level**[6]	Measure of readability.<br>Uses a modified Flesch Reading Ease formula to produce a grade-level score.<br>Evaluates which standard US school grade is needed to understand a text.	/	/	Level = (0.39 x average sentence length) + (11.8 x average number of syllables per word) - 15.59	Metric (interval)	-3.4 as the lowest grade level score in theory<br><br>no upper bound	>12: higher education level<br>≤12: grade level score corresponding to standard US school grades, in which 12 is the final year of high school.
**4. Entity related content**							
**Key fact score**	Evaluation of the video content according to key facts described in established clinical guidelines.	73 items addressing the categories: definition, symptoms, risk factors, evaluation, management, outcome, and risk of progression.<br>For specific items, compare Supplementary Table 2.	1: fully addressed<br>0.5: partially addressed<br>0: not addressed	Per item	Absolute number (%) of evaluated videos fully/partially/not addressing the respective item.	/	/
				Sum of scores for each of the 73 items.	Categorial (ordinal)	0-73	/
**5. Video related parameters**							
**Viewing rate**	Evaluates how often the video has been watched since upload.	/	/	views / days since upload	Metric (ratio)	/	/
**Engagement rate**	Evaluates the rate of engagement (likes, dislikes, comments) since upload.	/	/	(likes + dislikes + comments) / views	Metric (ratio)	/	/

HON, Health on the Net; JAMA, Journal of the American Medical Association.

1. Boyer C, Selby M, Appel RD. The Health On the Net Code of Conduct for medical and health web sites. *Stud Health Technol Inform* 1998; **52 Pt 2:** 1163-1166. e-pub ahead of print 1999/06/29;

2. Organisation H-N-G. Health On the Net. In, 2020.

3. Silberg WM, Lundberg GD, Musacchio RA. Assessing, controlling, and assuring the quality of medical information on the Internet: Caveant lector et viewor--Let the reader and viewer beware. *JAMA* 1997; **277**(15)**:** 1244-1245. e-pub ahead of print 1997/04/16;

4. Charnock D, Shepperd S, Needham G, Gann R. DISCERN: an instrument for judging the quality of written consumer health information on treatment choices. *J Epidemiol Community Health* 1999; **53**(2)**:** 105-111. e-pub ahead of print 1999/07/09; doi: 10.1136/jech.53.2.105

5. Flesch R. A new readability yardstick. *J Appl Psychol* 1948; **32**(3)**:** 221-233. e-pub ahead of print 1948/06/01; doi: 10.1037/h0057532

6. Kincaid JP, Fishburne RP, Rogers RL, Chissom BS. Derivation of new readability formulas (automated readability index, fog count, and flesch reading ease formula) for Navy enlisted personnel. *Chief of Naval Technical Training: Naval Air Station Memphis.* 1975; (Research Branch Report 8–75.).
