# Supplementary material for: Quality of Online Information on Multiple Myeloma Available for Laypersons
Source: Curr Oncol. 2022 Jun 27;29(7):4522–40. doi: 10.3390/curroncol29070358 (PMC9324394; doi:10.3390/curroncol29070358)
Supplement: Supplementary file 1 [file curroncol-29-00358-s001.zip › Supplementary_Table2.pdf]

Supplementary Table S2: Website and video content evaluation by MM key facts.

**Category**	**Item**	**Description**
Definition	Clonal PCs ≥ 10 %	Self-explanatory.
	Biopsy proven bony or extramedullary plasmacytoma	Biopsy proven local proliferation of clonal PCs.
	Hypercalcemia	Calcium > 2.75 mmol/l (>10.5 mg/dl) or > 0.25 mmol/l above the upper reference value.
	Renal insufficiency	Creatinine ≥ 2.0 mg/dl (> 173 µmol/l) or GFR < 40 ml/min.
	Anemia	Hemoglobin < 10.0 g/dl (< 6.21 mmol/l) or ≥ 2.0 g/l (> 1.24 mmol/l) below the lower reference value.
	Bone lesions	Detection of at least one bone lesion in imaging diagnostic.
	Clonal BM PCs ≥ 60 %	60 % or more PCs in BM.
	Involved:uninvolved serum FLC ratio ≥ 100	FLC ratio in serum ≥ 100 (involved/uninvolved FLC).
	> 1 focal lesion on MRI studies	More than 1 focal lesion with size of more than 1 cm detected in MRI.
	SLiM-CRAB	SLiM-CRAB (acronym): **s**ixty percent BM PCs, F**L**C ratio, lesions on **M**RI, hyper**c**alcemia, **r**enal insufficiency, **a**nemia, **b**one lesions.
Symptoms	Symptom-free	MM diagnosis might be an accidental finding.
	Bone pain	Bone pain caused by bone lesions.
	Fractures	Bone destruction may lead to pathological fractures.
	Fatigue	Tiredness and exhaustion caused by anemia.
	Infection susceptibility	Immunosuppression and consequently higher risk of infections caused by antibody deficiency and low leucocytes.
	B symptoms	B symptoms as fever, night sweat, loss of weight.
	Foaming urine	As a sign of increased protein in the urine (Bence-Jones-Proteinuria, albuminuria).
	Further symptoms of anemia	E.g. disturbance of memory, dizziness, angina pectoris, abdominal angina.
	Bleeding tendency	Caused by low platelet count.
	Amyloidosis-associated symptoms	Accumulation of abnormal monoclonal protein in different organs, with subsequent organ dysfunction leading to renal and cardiac insufficiency, neuropathy etc.
Risk factors	MGUS	The risk of progression from MGUS to MM is 1 %/year.
	Lymphoproliferative disease	Earlier diagnosis of MGUS might have been attributed to a lymphoproliferative disease.
	Age	Incidence increases with age.
	Etiology not known	The exact etiology is not known yet.
	People of color	Increased incidence in black people compared to Caucasian people.
	First degree relative	Increased risk in case of first-degree relatives diagnosed with MGUS.
	Genetic predisposition	There is a higher risk for people with a genetic predisposition.
	Further internal factors	E.g. obesity, chronic infections.
	External factors	E.g. radiation, pesticides, products of rubber production.
Evaluation	Medical history	Mandatory to obtain any relevant information on patient's health status.
	Clinical examination	Mandatory to assess any signs or symptoms of a medical condition.
	Differential blood count	Differentiation of white blood cells.
	Electrolytes	Sodium, potassium, calcium etc.
	Kidney retention parameters	Creatinine, GFR, urea.

	Total protein, albumin (S)	Quantity of total protein and albumin in serum.
	Protein electrophoresis (S)	Separates the serum protein components into five major fractions. Examines which globulin fraction is elevated.
	Immunofixation (S)	Assessment of the M protein type in serum.
	Immunofixation (U)	Assessment of the M protein type in urine.
	Igs (S)	Concentration of IgG, IgA, IgM in serum.
	FLCs (S)	Concentration of FLCs (kappa and lambda) in serum.
	24h-urine collection for protein quantification	Quantifies the urinary protein excretion per 24 h.
	LDH	Non-specific marker of cell turnover.
	Pro BNP	Normal level helps to rule out chronic heart failure.
	GPT	Evaluation of liver function.
	Beta2-microglobuline (S)	(Prognostic) marker in PC disorders, particularly MM.
	Low dose whole body CT without contrast agents	Detection of osteolyses and osteopenia, without contrast agents.
	Projection radiography NO standard of care	Projection radiography is no longer standard of diagnostics in PC disorders.
	MRI	Evaluation of diffuse BM infiltration, focal bone lesions and extramedullary manifestations.
	BM cytology	Assessment of extent of PC infiltration and status of other hematopoietic cells.
	BM histology	Assessment of extent of PC infiltration and status of other hematopoietic cells in the context of BM stroma.
	BM cytogenetics	Detection of chromosomal aberrations.
Prognostic factors	ISS	ISS classifies into 3 prognostic subgroups on the basis of serum albumin, beta2-microglobuline, LDH and cytogenetics.
	Cytogenetics	Cytogenetics (e.g. del(17p), t(4;14), t(14;16)) are important for prognostic significance.
	MRD	Although patient obtain complete remission in MRI, PET, molecular diagnostic tests and flow cytometry, there is still MRD detectable. MRD-negativity correlates with longer and progression-free survival.
Management	Osteoprotection	Osteoprotection is indicated, if at least one osteolytic lesion detectable (e.g. bisphosphonates, Denosumab). It reduces bone-related events.
	Radiotherapy	MM is radiation-sensitive, so radiation treats bone lesions and bone pain.
	Surgery	In order to stabilize the vertebral body with kyphoplasty.
	Corset	In order to stabilize the vertebral body.
	Transplant-eligible vs not transplant-eligible	Patients without comorbidities till the age of 75 years are transplant eligible, so they tolerate HD-chemotherapy.
	Induction therapy	4-6 bouts of VRD (Bortezomib, Lenalidomide, Dexamethasone) or VCD (Bortezomib, Cyclophosphamide, Dexamethasone) or VTD (Bortezomib, Thalidomide, Dexamethasone) or VD (Bortezomib, Dexamethasone).
	HD Melphalan SCT	Mobilization of stem cells with G-CSF, collecting stem cells, HD Melphalan (200 mg/m^2), transplantation of stem cells.
	Maintenance therapy	Maintenance therapy with Lenalidomide, Bortezomib or Thalidomide cause longer and progression-free survival.
	Treatment at relapse	It is mentioned that treatment at relapse is available.
	Continuous follow-up	Continuous follow-up for symptomatic patients: medical history, clinical examination, blood tests.
	Best supportive (palliative) care possible	It is mentioned that best supportive (palliative) care ist available.
	Specialized care center	Best treatment available in specialized care centers for hematological diseases.
	Clinical trials	For clinical advances and news in research.
	Rehabilitation	Patients should get rehabilitation procedures with oncological focus.

Supportive care	Psychosocial support	To help the patient handle their oncological situation.
	Nutrition	A well-balanced nutrition supports the patient to handle his disease.
	Sports	Sports and activities support the patient to handle his disease.
Outcome	Not curative	MM is not curative yet.
	Overall survival	Absolute: male 41 %, female 40 %; relative: male 48 %, female 45 %.

MM key facts were sourced from International Myeloma Working Group updated criteria for the diagnosis of multiple myeloma, Deutsche Gesellschaft für Hämatologie und Medizinische Onkologie e. V. (DGHO) on MM, and “Multiple myeloma: EHA-ESMO Clinical Practice Guidelines for diagnosis, treatment and follow-up”.[1-3]

BM, bone marrow; BNP, brain natriuretic peptide; CT, computer tomography; FLC, free light chain; G-CSF, granulocyte colony-stimulating factor; GFR, glomerular filtration rate; GPT, glutamate pyruvate transaminase; HD, high dose; Ig, immunoglobulin; ISS, international staging system; LDH, lactate dehydrogenase; MGUS, monoclonal gammopathy of undetermined significance; MM, multiple myeloma; MRD, minimal residual disease; MRI, magnetic resonance imaging; PC, plasma cell; PET, positron emission tomography; S, serum; SCT, stem cell transplantation; U, urine.

1. Rajkumar SV, Dimopoulos MA, Palumbo A, Blade J, Merlini G, Mateos MV *et al.* International Myeloma Working Group updated criteria for the diagnosis of multiple myeloma. *Lancet Oncol* 2014; **15**(12)**:** e538-548. e-pub ahead of print 2014/12/03; doi: 10.1016/S1470-2045(14)70442-5

2. Wörmann B, Driessen C, Einsele H, Goldschmidt H, Gunsilius E, Kortüm M *et al.* Multiples Myelom. In: DGHO, Onkopedia Leitlinien, 2018.

3. Dimopoulos MA, Moreau P, Terpos E, Mateos MV, Zweegman S, Cook G *et al.* Multiple Myeloma: EHA-ESMO Clinical Practice Guidelines for Diagnosis, Treatment and Follow-up. *Hemasphere* 2021; **5**(2)**:** e528. doi: 10.1097/HS9.0000000000000528
