# Supplementary material for: Quality of Online Information on Multiple Myeloma Available for Laypersons
Source: Curr Oncol. 2022 Jun 27;29(7):4522–40. doi: 10.3390/curroncol29070358 (PMC9324394; doi:10.3390/curroncol29070358)
Supplement: Supplementary file 1 [file curroncol-29-00358-s001.zip › Supplementary_Table3.pdf]

Supplementary Table S3. Search results for websites and videos.

Health information source	A. Websites			B. Videos
**Search engine/platform**	**Google**	**Bing**	**Yahoo**	**YouTube**
**Initial search, n (%)**	100 (100)	100 (100)	100 (100)	50 (100)
**Duplicates within each search engine, n (%)**	21 (21.0)	46 (46.0)	59 (59.0)	/
**Eligibility criteria not met, n**				
No relevance to MM	22	13	16	1
Access required	3	3	2	0
Other language	0	0	0	0
Scientific article	1	0	0	1
Duplicate	/	/	/	3
∑ (%)	26 (26.0)	16 (16.0)	18 (18.0)	5 (10.0)
**Included websites for search engine, n (%)**	53 (53.0)	38 (38.0)	23 (23.0)	/
**Overlap, n**				
Google/Bing		9		/
Bing/Yahoo		2		/
Yahoo/Google		1		/
Google/Bing/Yahoo		19		/
**Overall unique, n (%)**		**63 (100)**		**45 (90.0)**

MM, multiple myeloma.
