# Supplementary material for: Quality of Online Information on Multiple Myeloma Available for Laypersons
Source: Curr Oncol. 2022 Jun 27;29(7):4522–40. doi: 10.3390/curroncol29070358 (PMC9324394; doi:10.3390/curroncol29070358)
Supplement: Supplementary file 1 [file curroncol-29-00358-s001.zip › Supplementary_Table4.pdf]

Supplementary Table S4. Characterization of websites by search engine.

Search engine	Google	Bing	Yahoo	*P* value
**Websites, n**	53	38	23	
**Website category, n (%)**				
Scientific/governmental	1 (1.9)	1 (2.6)	1 (4.3)	
Foundation/advocacy	26 (49.1)	21 (55.3)	11 (47.8)	
News/media	10 (18.9)	5 (13.2)	4 (17.4)	0.955[a]
Industry/for profit	16 (30.2)	11 (28.9)	7 (30.4)	
Personal commentary/blog	0 (0.0)	0 (0.0)	0 (0.0)	
**Paid/promoted, n (%)**	9 (17.0)	4 (10.5)	2 (8.7)	0.520
**Host continent, n (%)**				
Europe	1 (1.9)	1 (2.6)	0 (0)	/
North America	52 (98.1)	37 (97.4)	23 (100)	
South America	0 (0.0)	0 (0.0)	0 (0.0)	
Asia	0 (0.0)	0 (0.0)	0 (0.0)	
Australia	0 (0.0)	0 (0.0)	0 (0.0)	
Africa	0 (0.0)	0 (0.0)	0 (0.0)	
Antarctica	0 (0.0)	0 (0.0)	0 (0.0)	
**HON foundation certificate**				
Valid certificate, n (%)	8 (15.1)	9 (23.7)	6 (26.1)	0.579
**JAMA score**				
Median (range)	3 (1-4)	3 (1-4)	3 (1-4)	0.368
**Flesch Reading Ease Score**				
Mean (SD)	39 (9)	41 (10)	40 (11)	0.802
**Flesch Kincaid Grade Level**				
Mean (SD)	13 (2)	12 (2)	12 (2)	0.788
**Sum DISCERN score**				
All websites, median (range)	42 (17-68)	42 (22-68)	41 (22-68)	0.643
Top 10 website hits, median (range)	46 (27-61)	44 (27-61)	42 (22-61)	0.871
**Sum key fact score**				
All websites, median (range)	26 (4-54)	25 (5-54)	24 (5-54)	0.957
Top 10 website hits, median (range)	36 (5-53)	21 (5-53)	30 (11-53)	0.918
**Misleading/wrong facts**				
Websites with misleading/wrong facts, n (%)	10 (18.9)	8 (21.1)	5 (21.7)	0.935
Overall identified wrong facts, n	13	11	6	

[a] Foundation/advocacy versus news/media reports versus industry/for profit. HON, Health on the Net; JAMA, Journal of the American Medical Association; SD, standard deviation.
