# Supplementary material for: Quality of Online Information on Multiple Myeloma Available for Laypersons
Source: Curr Oncol. 2022 Jun 27;29(7):4522–40. doi: 10.3390/curroncol29070358 (PMC9324394; doi:10.3390/curroncol29070358)
Supplement: Supplementary file 1 [file curroncol-29-00358-s001.zip › Supplementary_Table5.pdf]

Supplementary Table S5: DISCERN score for websites and videos.

Health information source	A. Websites		B. Videos
**Overall unique websites/videos, n (%)**	63 (100)		45 (100)
**Section 1: Is the publication reliable? (item 1-8), median (range)**			
Explicit aims	3 (1-4)		3 (1-4)
Aims achieved	3 (1-4)		3 (2-4)
Relevance to patients	3 (1-5)		3 (1-4)
Sources of information	1 (1-5)		1 (1-5)
Currency (date) of information	3 (1-5)		1 (1-5)
Bias and balance	3 (1-5)		1 (1-5)
Additional sources of information	2 (1-5)		1 (1-3)
Reference to areas of uncertainty	1 (1-4)		1 (1-3)
Overall section 1	*19 (9-35)*		*14 (9-30)*
**Section 2: How good is the quality of information on treatment choices? (item 9-15), median (range)**			
How treatment works	2 (1-5)		1 (1-4)
Benefits of treatment	3 (1-5)		3 (1-5)
Risks of treatment	1 (1-5)		1 (1-5)
No treatment options	2 (1-5)		1 (1-4)
Quality of life	2 (1-5)		1 (1-3)
Other treatment options	5 (1-5)		1 (1-5)
Shared decision making	2 (1-5)		1 (1-5)
Overall section 2	*17 (7-34)*		*12 (7-23)*
**Section 3: Overall rating of the publication (item 16), median (range)**			
Overall quality	3 (1-5)		2 (1-4)
