# Supplementary material for: Quality of Online Information on Multiple Myeloma Available for Laypersons
Source: Curr Oncol. 2022 Jun 27;29(7):4522–40. doi: 10.3390/curroncol29070358 (PMC9324394; doi:10.3390/curroncol29070358)
Supplement: Supplementary file 1 [file curroncol-29-00358-s001.zip › Supplementary_Table6.pdf]

Supplementary Table S6. Misleading and wrong facts on websites and videos.

Health information source	A. Websites		B. Videos
**Overall unique websites/videos, n (%)**	63 (100)		45 (100)
**Websites with misleading/wrong facts, n (%)**	10 (15.9)	**Videos with misleading/wrong facts, n (%)**	1 (2.2)
By website category, n		By video category, n	
Scientific/governmental	1	Academic institution	0
Foundation/advocacy	2	Governmental organization	0
News/media	3	News/media	0
Industry/for profit	4	Industry/for profit	0
Personal commentary/blog	0	Independent medical professional	1
		Independent non-medical user	0
**Misleading/wrong facts**			
Overall, n	15		1
By key fact category, n (%)			
Definition	3 (20.0)		0 (0.0)
Symptoms	0 (0.0)		0 (0.0)
Risk factors	1 (6.7)		0 (0.0)
Evaluation	2 (13.3)		1 (2.2)
Prognostic factors	1 (6.7)		0 (0.0)
Management	8 (53.3)		0 (0.0)
Supportive care	0 (0.0)		0 (0.0)
Outcome	0 (0.0)		0 (0.0)
